# Steric regulation of CRISPR/Cas12a *trans*-cleavage kinetics via split-activator extensions

**DOI:** 10.1093/nar/gkaf1535

**Published:** 2026-01-14

**Authors:** Jianhong Zhang, Xin He, Jing Huang, Cheng Cheng, Guowei He, Ruili Xia, Jun Yang, Jianmei Chen, Lirong Guo, Debing Xiang, Feng Li, Jing Shi, Pu Li

**Affiliations:** Department of Clinical Laboratory, Chongqing University Jiangjin Hospital, School of Medicine, Chongqing University, Chongqing 402260, China; Department of Clinical Laboratory, Chongqing University Jiangjin Hospital, School of Medicine, Chongqing University, Chongqing 402260, China; Office of Hospital Administration, Chongqing University Jiangjin Hospital, School of Medicine, Chongqing University, Chongqing 402260, China; Office of Hospital Administration, Chongqing University Jiangjin Hospital, School of Medicine, Chongqing University, Chongqing 402260, China; Department of Clinical Laboratory, Chongqing University Jiangjin Hospital, School of Medicine, Chongqing University, Chongqing 402260, China; Department of Clinical Laboratory, Chongqing University Jiangjin Hospital, School of Medicine, Chongqing University, Chongqing 402260, China; Department of ICU, Chongqing University Jiangjin Hospital, School of Medicine, Chongqing University, Chongqing 402260, China; Department of ICU, Chongqing University Jiangjin Hospital, School of Medicine, Chongqing University, Chongqing 402260, China; Department of Pediatrics, Chongqing University Jiangjin Hospital, School of Medicine, Chongqing University, Chongqing 402260, China; Office of Hospital Administration, Chongqing University Jiangjin Hospital, School of Medicine, Chongqing University, Chongqing 402260, China; Office of Hospital Administration, Chongqing University Jiangjin Hospital, School of Medicine, Chongqing University, Chongqing 402260, China; Department of Laboratory Medicine, The First Affiliated Hospital of Chongqing Medical University, Chongqing 400016, China; Department of Clinical Laboratory, Chongqing University Jiangjin Hospital, School of Medicine, Chongqing University, Chongqing 402260, China

## Abstract

Clustered regularly interspaced short palindromic repeats (CRISPR)/Cas12a holds substantial promise for molecular diagnostics, yet its rapid and uncontrolled activation often results in background leakage and disrupts the coordination of upstream reaction modules. Here, we established a steric-regulation framework that enables predictable tuning of Cas12a *trans*-cleavage kinetics through rationally engineered extensions on split activators. Systematic analysis of extension orientation, length, and hybridization state revealed quantitative and direction-dependent rules governing steric control of activator assembly and Cas12a activation. Guided by these insights, we integrated the sterically regulated split activator into an entropy-driven DNA circuit to construct a fully one-pot cascaded detection system. The engineered steric barriers effectively suppressed premature activation and established precise kinetic matching between the DNA circuit and Cas12a. The resulting platform achieved a detection limit of 1.24 pM for microRNA-21 and demonstrated high fidelity. This work defines a predictable steric-gating mechanism for Cas12a activation and delivers a nucleic-acid-only regulatory module that can be incorporated into diverse CRISPR architectures, supporting the development of robust, leakage-resistant one-pot diagnostic systems.

## Graphical Abstract

**Graphical Abstract:**
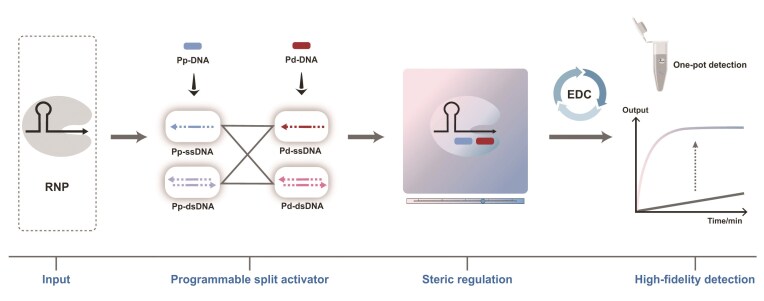

## Introduction

Clustered regularly interspaced short palindromic repeats (CRISPR) and their associated proteins (Cas) originate from the adaptive immune systems of prokaryotes, enabling them to recognize and cleave invading foreign genetic material [1–5]. Cas12a, as an effector protein of the class II type V CRISPR system, is a CRISPR RNA (crRNA)-guided DNA endonuclease [6, 7]. It recognizes a T-rich protospacer adjacent motif (PAM) on a target double-stranded DNA (dsDNA), triggering local duplex unwinding and R-loop formation [8, 9]. Subsequently, it utilizes the RuvC catalytic domain to sequentially cleave the target strand (TS) and nontarget strand (NTS), completing the *cis-*cleavage process. Once activated, Cas12a also possesses a nonspecific *trans*-cleavage activity that cleaves single-stranded DNA (ssDNA) [10–12]. Based on these characteristics, Cas12a shows broad application potential in fields such as gene editing, molecular diagnostics, and transcriptional regulation [13–17].

However, with the rapid expansion of CRISPR/Cas12a in molecular diagnostics, the lack of spatiotemporal control over its activation process is becoming increasingly prominent [18, 19]. Current regulatory strategies primarily focus on crRNA modification, including light-controlled crRNA activation [20], circular crRNA [21], methylated crRNA [22], G-quadruplex-guided crRNA [23], strand displacement crRNA [24], engineered crRNA [25], and tandem crRNA [26], among others. However, these methods suffer from limitations such as complex design, potential biosafety risks, or insufficient operational simplicity, and they struggle to achieve continuous regulation of activation intensity and timing [27–29]. Furthermore, enzyme-dependent cascaded systems employing recombinase polymerase amplification [30] or loop-mediated isothermal amplification [31] frequently suffer from reaction incompatibility and kinetic misalignment. In contrast, isothermal, enzyme-free amplification strategies like hybridization chain reaction [32] or catalytic hairpin assembly [33] operate under mild conditions but suffer from low signal yield, making it difficult to meet the demands of short-term clinical diagnostics. Therefore, developing a versatile, molecular programming-based regulatory strategy that allows for programmable modification of activators other than crRNA and requires no external stimulation has become a key to promoting the clinical translation of CRISPR technology [34, 35].

To address these bottlenecks, this study established a steric-regulated split activation strategy. Tunable spatial extensions introduced at the 5′ or 3′ terminus generate programmable steric hindrance that modulates the interaction geometry within the split-activator–crRNA–Cas12a complex. This controllable steric environment enabled graded control of Cas12a *trans*-cleavage kinetics, allowing for precise tuning of both the activation rate and cleavage magnitude. We first examined the intrinsic activation capacity of split ssDNA activators and then systematically investigated how extension length, directionality, and structural rigidity (ssDNA versus dsDNA) reshape steric constraints to govern Cas12a cleavage dynamics. To rigorously mimic the suboptimal targeting conditions commonly encountered in biological samples, kinetic analyses were performed primarily under noncanonical PAM contexts, with the canonical PAM included for comparison. Guided by these steric-regulation principles, we incorporated the optimized split activator into an entropy-driven DNA circuit (EDC) to construct a leakage-suppressed, one-pot cascaded detection system. Using microRNA (miRNA)-21 as a model analyte, we validated the system’s detection sensitivity, linear range, and specificity, confirming its improved signal fidelity and overall performance. In summary, this steric-regulation framework provides a molecularly programmable approach for fine control of Cas12a activity, supporting the development of high-fidelity CRISPR diagnostic platforms and adaptable CRISPR-based molecular circuits.

## Materials and methods

### Reagents and instruments

The DNA oligonucleotides and RNA used in this study were synthesized and high pressure (or high performance) liquid chromatography-purified by Shanghai Sangon Biotech Co., Ltd. (Shanghai, China). Detailed sequences and modification information are listed in Supplementary Table S1. Lba Cas12a (Cpf1, 100 μM) and 10× rCutSmart buffer (50 mM potassium acetate, 20 mM Tris-acetate, 10 mM magnesium acetate, 100 μg/ml recombinant albumin, pH 7.9) were purchased from New England Biolabs (Beijing, China). Tris-EDTA (TE) buffer [10 mM Tris–HCl, 1 mM ethylenediaminetetraacetic acid (EDTA), pH 8.0], Diethyl pyrocarbonate (DEPC)-treated water (RNase-free), dithiothreitol (DTT), magnesium chloride hexahydrate (MgCl₂·6H₂O), and sodium chloride (NaCl) were purchased from Shanghai Sangon Biotech Co., Ltd. (Shanghai, China).

Fluorescence signal monitoring was performed using a real-time fluorescence quantitative polymerase chain reaction (PCR) system FQD-96A (BIOER, China). The reaction temperature was set at 37°C, the green fluorescence channel was selected, and the gain was set to the default value. Oligonucleotide annealing reactions were also performed on this PCR instrument.

### Sample preparation

DNA oligonucleotides were dissolved in TE buffer (10 mM Tris–HCl, 1 mM EDTA, pH 8.0), and RNA oligonucleotides were dissolved in DEPC water. DNA complexes were assembled in TE/Mg^2^⁺ buffer [10 mM Tris–HCl, 1 mM EDTA, 12.5 mM MgCl_2_, pH 8.0] by mixing corresponding complementary single strands in equimolar concentrations. The annealing process was performed on a PCR instrument: heated at 95°C for 5 min, and then ramped down to 20°C at a rate of 0.1°C/s. After annealing, the complexes were stored at 4°C for later use.

### Analysis of Cas12a *trans*-cleavage activity mediated by split activators

To evaluate how different split-activator architectures regulate Cas12a *trans*-cleavage activity, we examined the performance of various ssDNA and dsDNA split activators both individually and in their assembled mixed states. Each reaction (20 μl) contained 20 nM ribonucleoprotein (RNP), 10 nM of the corresponding split-activator components, 250 nM reporter, 1× rCutSmart buffer, and 1 mM DTT, with the remaining volume supplemented using TE/Mg^2^⁺ buffer. Reactions were carried out at 37°C, and fluorescence kinetics were recorded in real time using a quantitative PCR fluorescence reader.

### Construction and performance evaluation of the one-pot EDC–Cas12a cascaded system

To investigate kinetic matching within the cascaded reaction, we constructed and compared three activation strategies: canonical activation, conventional split activation, and steric-regulated split activation. A typical one-pot reaction (20 μl) contained 100 nM three-strand substrate complex, 100 nM fuel strand, 10 nM miRNA-21, 20 nM RNP, 250 nM reporter, 1× rCutSmart buffer, and 1 mM DTT. For strategies involving split activation, 100 nM of the fixed-end split strand was preincubated with RNP to form the initial complex, while the output strand generated by the EDC cycle served as the complementary activating component.

After identifying the optimal steric-regulated split-activator design, we further evaluated the analytical performance of the optimized one-pot system. The evaluation mixture (20 μl) consisted of 100 nM S0’–S1–S2’, 100 nM F’, 80 nM RNP, 100 nM Pd-ss11_DE3, 250 nM reporter, 1× rCutSmart buffer, and 1 mM DTT. By introducing various concentrations of miRNA-21 or interfering sequences and monitoring fluorescence at 37°C in real time, we assessed the sensitivity, linear range, and specificity of the cascaded system.

### Normalization of fluorescence signals

Fluorescence data were normalized to quantify the regulatory effects of different split-activator designs on Cas12a activity. The relative cleavage efficiency was calculated using the equation: % cleaved = *F*/*F*_PC_ × 100%. Here, *F* denoted the fluorescence intensity of the experimental group, and *F*_PC_ represented the plateau fluorescence of the Positive Control (PC), defined as the reaction containing the canonical full-length TS that yielded maximal *trans*-cleavage activity. The Negative Control represented the baseline signal and was obtained from reactions containing all components except the activator strands. Because different regulatory strategies originated from distinct initial structural states, the Control (C) condition was defined operationally for each strategy as the unmodified baseline design prior to introducing steric regulatory elements. Depending on the specific strategy being evaluated, the C condition corresponded to a native ssDNA split pair, a dsDNA-anchored construct, or a minimally extended variant. In each case, the C condition served as the internal reference for evaluating the quantitative impact of subsequent structural modifications. By normalizing all experimental signals to the canonical full-length activator (PC) and referencing each strategy against its internal baseline (C), this framework enabled consistent comparison of relative activation efficiencies across distinct steric-regulated designs.

### Statistical analysis

All quantitative data were obtained from at least three independent experimental replicates and are presented as mean ± standard deviation (SD), unless otherwise noted. Data preprocessing, curve fitting, kinetic parameter extraction, and signal-to-background calculations were performed using Origin 2024 (OriginLab, USA). Graphs and numerical summaries were generated using Origin 2024 and GraphPad Prism 9 (GraphPad Software, USA).

## Results and discussion

### Screening of low-background split activators

**Figure 1.**
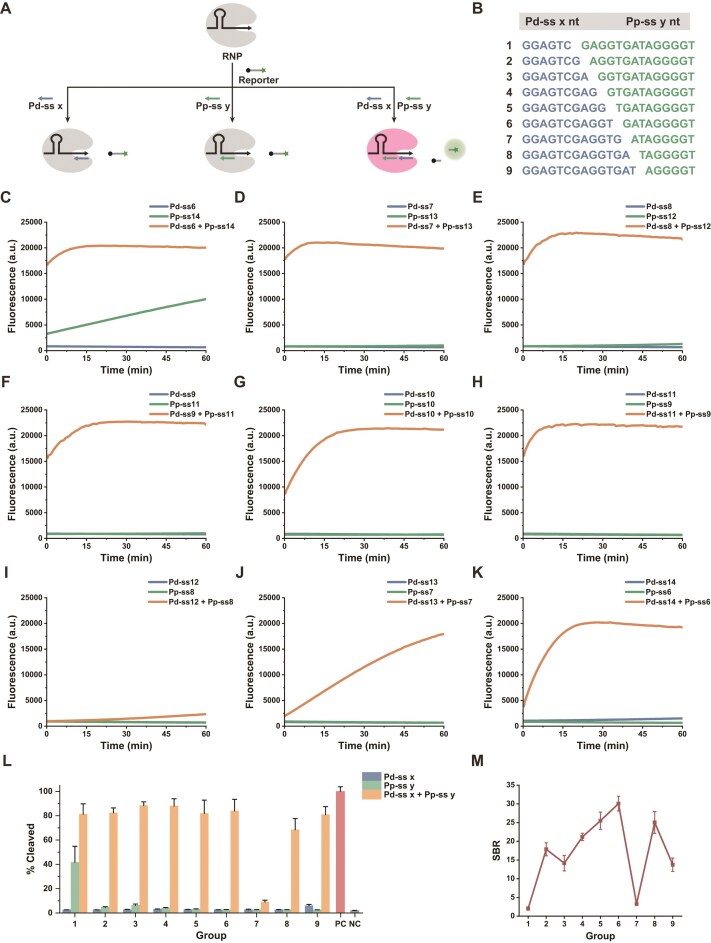
Screening of split ssDNA activators and identification of low-leakage combinations. (**A**) Schematic of Cas12a activation by split ssDNA. (**B**) Design of nine split-activator pairs generated by varying the breakpoint within the spacer-complementary region. (**C**–**K**) Real-time fluorescence kinetics for the nine split-activator groups. (**L**) Activation efficiency at 60 min. (**M**) Signal-to-background ratio (SBR) analysis at 60 min. Data are presented as mean ± SD (*n* = 3).

To construct a programmable and ultra-low-background Cas12a regulatory platform, the primary task is to identify a set of split activators that remain silent in isolation but efficiently activate Cas12a upon assembly. Although a single short ssDNA is typically insufficient to induce the conformational changes required for *trans*-cleavage, the correct assembly of two complementary split strands guided by crRNA can effectively restore enzymatic activity (Fig. 1A). However, the choice of the split site fundamentally dictates the binding energy of the fragments, the thermodynamic stability of the assembly, and the risk of potential single-strand leakage. To this end, we systematically designed nine sets of ssDNA combinations with varying split topologies based on the complementary sequence of the crRNA spacer (designated as Pd-ss x and Pp-ss y, Fig. 1B) and screened their activation kinetics. As shown in Fig. 1C–K, the split site exerted a decisive impact on system performance. In combinations with longer Pp fragments (Groups 1–3), significant single-strand leakage was observed. Notably, in Group 1 (Fig. 1C), the addition of single-stranded Pp-ss14 alone induced a marked rise in fluorescence signal, indicating that the overly long single-stranded fragment is capable of independently inducing significant background cleavage activity. Conversely, when the Pd fragment was longer while the Pp fragment was too short (Groups 7 and 8), the single-strand leakage was effectively suppressed, but the overall activation efficiency upon mixing was significantly compromised (Fig. 1I–J). For Group 9 (Fig. 1K), although its mixed activation efficiency recovered to a relatively high level, the overly long Pd fragment (14 nt) led to the reappearance of single-strand background signal. In contrast, Groups 4, 5, and 6 achieved a favorable balance between activation activity and background noise (Fig. 1F–H).

To identify the optimal candidate among these preferred groups, performance was quantitatively evaluated using the SBR (Fig. 1M), calculated by dividing the peak fluorescence of the mixed assembly by the maximum background leakage generated by either single-stranded component. This evaluation strategy benchmarks against the system’s most severe potential leakage, reflecting true activation efficacy. Given the marginal differences among the three groups within the standard reaction time, we further performed a 180-min long-term kinetic validation (Supplementary Fig. S1). The results showed that the single-stranded components of Groups 4 and 5 exhibited slight signal drift over time, suggesting a risk of metastable binding; only Group 6 maintained its single-stranded components at an extremely low baseline level throughout the monitoring window, achieving a significantly higher ratio compared to other combinations. Based on this rigorous screening, we established Pd-ss11 and Pp-ss9 as the optimal split-activator combination. This combination nearly eliminates basal leakage mediated by single components while retaining high assembly-dependent activation capacity, providing an ideal low-background structural foundation for the subsequent introduction of programmable steric regulation via terminal extensions.

### Steric regulation of Cas12a kinetics in fixed single-stranded scaffolds

To elucidate the molecular basis underlying kinetic regulation, we first employed flexible ssDNA as the anchoring scaffold of the split-activator system, introducing terminal extensions on the partner strand to impose programmable steric hindrance. Using Pd-ss11 and Pp-ss9 as the core split modules, we fixed one strand and progressively extended the 5′ end, 3′ end, or both ends (3–12 nt) of the other strand to systematically evaluate how distinct spatial configurations influence Cas12a activation kinetics. For clarity, extended strands are designated by appending 5′E (5′-end extension), 3′E (3′-end extension), or DE (dual-end extension) followed by length to the core name, with ss and ds denoting single-stranded and double-stranded forms, respectively.

**Figure 2.**
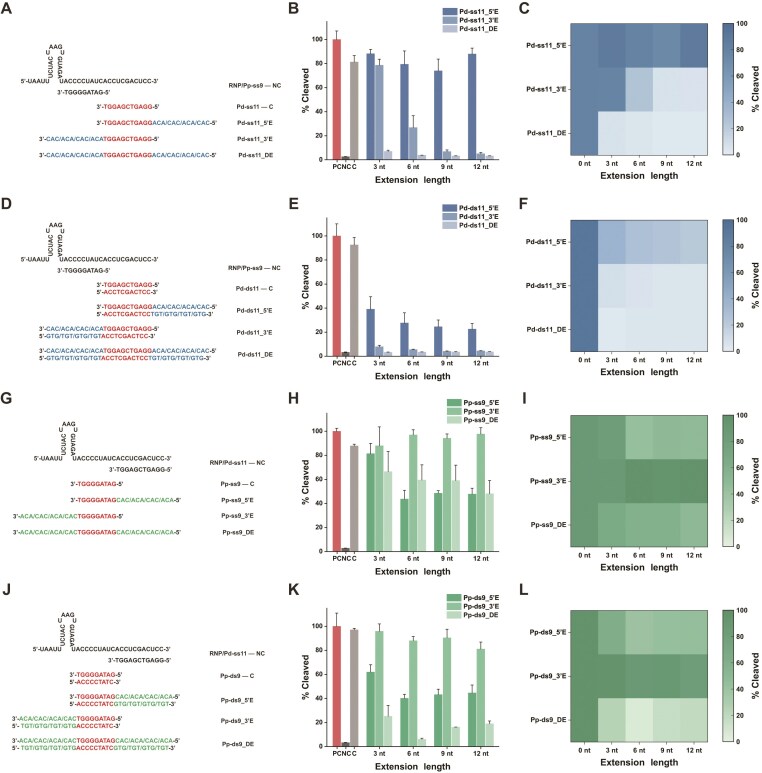
Steric regulation using a fixed single-stranded scaffold. With fixed Pp-ss9 and Pd-ss11 bearing ssDNA extensions. (**A**) Schematic, (**B**) activation efficiency, and (**C**) activity heatmap. With fixed Pp-ss9 and Pd-ds11 bearing rigid dsDNA extensions. (**D**) Schematic, (**E**) efficiency, and (**F**) heatmap. With fixed Pd-ss11 and Pp-ss9 bearing ssDNA extensions. (**G**) Schematic, (**H**) efficiency, and (**I**) heatmap. With fixed Pd-ss11 and Pp-ds9 bearing rigid dsDNA extensions. (**J**) Schematic, (**K**) efficiency, and (**L**) heatmap. All quantitative data represent relative *trans*-cleavage activity at 30 min. Data are presented as mean ± SD (*n* = 3).

We first examined the configuration in which Pp-ss9 was fixed while Pd-ss11 was varied (Fig. 2A). Real-time fluorescence kinetic revealed pronounced directional sensitivity (Supplementary Fig. S2). 3′-end extensions markedly suppressed both the activation rate and signal amplitude in a length-dependent manner, whereas 5′-end extensions had only minimal impact (Fig. 2B and C). Dual-end extensions produced additive inhibitory effects, substantially stronger than those of single-end extensions, indicating that the 3′ terminus of Pd-ss11 represents a sterically sensitive hotspot. When these flexible ssDNA extensions were replaced with rigid dsDNA extensions (Fig. 2D), steric hindrance effects were further amplified. Kinetic monitoring (Supplementary Fig. S3) showed that 3′ dsDNA extensions sharply reduced Cas12a activity, and dual dsDNA extensions nearly abolished activation, demonstrating the physical additivity of dual-end steric obstruction (Fig. 2E and F).

We next evaluated the complementary anchoring configuration, in which Pd-ss11 was fixed and Pp-ss9 was extended with terminal ssDNA additions (Fig. 2G). Under ssDNA extensions, the system exhibited a mirror-like regulatory pattern (Supplementary Fig. S4). 5′-end extensions acted as the dominant inhibitory factor, causing pronounced delays in activation, whereas 3′-end extensions did not suppress activity and instead maintained or increased it (Fig. 2H and I), suggesting that this terminus may adopt local conformations favorable for R-loop formation. Nonetheless, dual-end extensions (Pp-ss9_DE) still produced marked inhibition due to the presence of the sensitive 5′-end steric site, demonstrating the robustness of dual-end strategies even when one terminus is intrinsically tolerant. When ssDNA extensions were replaced with rigid dsDNA extensions (Fig. 2J), steric suppression was further strengthened (Supplementary Fig. S5). 5′ dsDNA extensions significantly delayed activation, while dual dsDNA extensions exerted the strongest cumulative blocking effect, reducing the fluorescence signal and overriding the noninhibitory nature of the 3′ terminus (Fig. 2K and L). To assess whether these steric effects introduced by the duplex scaffold were influenced by PAM specificity, we evaluated a representative combination (fixed Pd-ss11 with Pp-ds9_DE6) across four PAM variants (canonical TTTA and three noncanonical sequences; Supplementary Fig. S6). Although PAM identity shaped the intrinsic activation rate and signal magnitude, the fundamental steric regulatory trend remained unchanged, indicating that steric modulation functions as a physical control layer independent of sequence-specific recognition.

Together, these comparative analyses establish three fundamental principles governing steric regulation within ssDNA-anchored systems: (i) directional asymmetry and length dependence, with Pd-3′ and Pp-5′ identified as steric hotspots; (ii) dual-end extensions generate additive steric barriers, enabling effective inhibition even when one terminus is nonsensitive; and (iii) rigid structures amplify steric obstruction, providing stronger kinetic barriers than flexible ssDNA. These findings demonstrate that Cas12a activation kinetics can be precisely regulated by rationally designing the geometry, physical rigidity, and extension length of split-activator termini.

### Amplified steric regulation in fixed rigid double-stranded scaffolds

Having established the principles of steric regulation in ssDNA scaffolds, we next investigated how steric effects evolve when the anchoring strand is replaced by a more rigid dsDNA. Short duplexes (9 or 11 bp) exhibit insufficient thermal stability at 37°C, making unextended Pd-ds11 or Pp-ds9 unable to maintain rigid conformations. To obtain stable duplex anchoring points without introducing background activation, we introduced a necessary 6-nt extension at the nonsensitive terminus of the fixed strand, as guided by the principles derived above, generating Pd-ds11_5′E6 and Pp-ds9_3′E6. NUPACK simulations confirmed that both designs formed highly stable duplexes under reaction conditions (Supplementary Fig. S7), ensuring that observed kinetic differences predominantly arose from the intended steric manipulations rather than scaffold dissociation.

**Figure 3.**
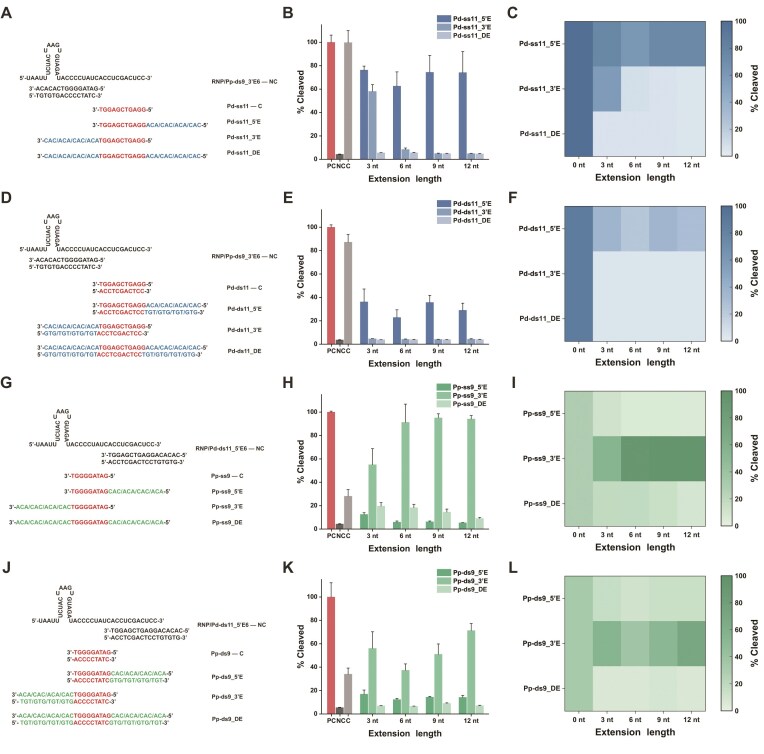
Amplified steric regulation using a fixed rigid double-stranded scaffold. With fixed Pp-ds9_3′E6 and Pd-ss11 bearing ssDNA extensions. (**A**) Schematic, (**B**) activation efficiency, and (**C**) activity heatmap. With fixed Pp-ds9_3′E6 and Pd-ds11 bearing rigid dsDNA extensions. (**D**) Schematic, (**E**) efficiency, and (**F**) heatmap. With fixed Pd-ds11_5′E6 and Pp-ss9 bearing ssDNA extensions. (**G**) Schematic, (**H**) efficiency, and (**I**) heatmap. With fixed Pd-ds11_5′E6 and Pp-ds9 bearing rigid dsDNA extensions. (**J**) Schematic, (**K**) efficiency, and (**L**) heatmap. All quantitative data represent relative *trans*-cleavage activity at 30 min. Data are presented as mean ± SD (*n* = 3).

We first anchored the rigid Pp-ds9_3′E6 scaffold and introduced extensions on Pd-ss11 (Fig. 3A). Compared with the ssDNA-anchored system, this configuration exhibited markedly enhanced steric sensitivity (Supplementary Fig. S8). As shown in Fig. 3B and C, 3′-end ssDNA extensions on Pd-ss11 strongly suppressed Cas12a activation in a sharply length-dependent manner, and dual-end extensions produced additive inhibitory effects. When Pd ssDNA extensions were replaced with rigid dsDNA extensions (Fig. 3D and Supplementary Fig. S9), suppression became even stronger. 3′ dsDNA extensions nearly extinguished fluorescence signals, whereas dual-end dsDNA extensions served as an almost complete physical “blocker,” reducing activity to background levels across all tested lengths (Fig. 3E and F), demonstrating synergistic steric exclusion contributed by the rigid scaffold and rigid extensions.

We next analyzed the complementary configuration, where Pd-ds11_5′E6 was fixed and Pp-ss9 was extended (Fig. 3G). In this arrangement, Pp-ss9_5′ extensions remained the dominant inhibitory contributor (Fig. 3H and I, and Supplementary Fig. S10). Conversely, Pp-ss9_3′ extensions did not exhibit inhibition across the examined lengths and instead increased activity, again indicating high conformational tolerance at this terminus. As a result, Pp-ss9_DE displayed intermediate inhibition, with the noninhibitory 3′ end partially buffering the 5′ steric effect. However, when Pp ssDNA extensions were replaced with rigid dsDNA (Fig. 3J and Supplementary Fig. S11), steric suppression regained full dominance. 5′ dsDNA extensions markedly diminished activity, and dual-end dsDNA extensions effectively overcame the inherent 3′-end tolerance, yielding near-complete inhibition of Cas12a activation (Fig. 3K and L). To assess the generality of rigidity-amplified steric regulation, we examined the fully rigid combination (Pd-ds11_5′E6 with Pp-ds9_DE6) across different PAM variants (Supplementary Fig. S12). PAM identity continued to modulate activation strength; however, relative to ssDNA systems, introducing a second rigid anchor substantially reduced fluorescence across all PAM contexts, indicating that duplex anchoring restricts molecular degrees of freedom to generate PAM-independent global inhibition.

Based on these systematic investigations, we established the following universal quantitative principles of steric regulation: (i) strong directional specificity, with Pd-3′ and Pp-5′ serving as key steric hotspots; (ii) regulation strength can be continuously tuned by extension rigidity and length, with rigid duplex scaffolds markedly amplifying steric effects and lowering inhibitory thresholds; (iii) conformational tolerance at specific termini can be effectively overcome by rigid duplex extensions; and (iv) dual rigid-end extensions integrate steric constraints to produce a robust “shutdown” mode, suppressing Cas12a activation to near-background levels. These principles constitute a programmable “structural rule” framework that provides clear design guidance for engineering high-performance Cas12a systems with rationally tailored steric regulation.

### Steric-regulated split activation enables robust one-pot cascaded detection

Nucleic acids, with their high programmability, predictable thermodynamic properties, and low synthesis costs, have become ideal components for molecular nanotechnology [36, 37]. These characteristics have driven the development of various self-assembled structures and dynamic reaction networks, enabling precise spatiotemporal control through mechanisms such as strand displacement and extension [38, 39]. As a typical nonenzymatic amplification strategy, the EDC can achieve efficient signal conversion from TSs to output strands through strand-displacement reactions, making it an ideal choice for molecular diagnostics [40, 41]. Therefore, we further explored whether it is possible to achieve precise control of CRISPR/Cas programmable activation, without external stimuli or additional modifications, solely relying on the inherent programmability of nucleic acids. This would allow it to be effectively isolated in the temporal domain from the upstream reaction network, thereby establishing an efficient, low-background one-pot cascaded system.

**Figure 4.**
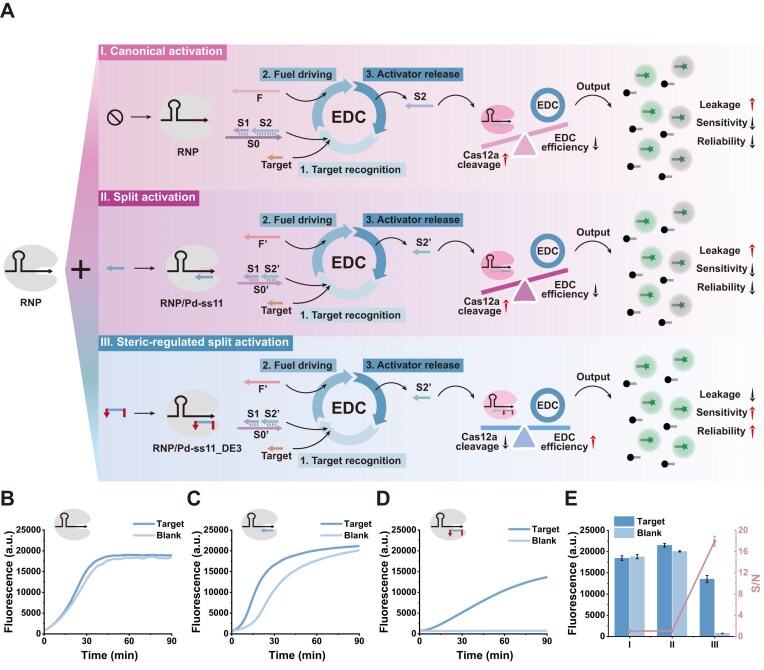
Comparison of activation strategies and construction of the one-pot EDC–Cas12a cascade. (**A**) Schematics of three activation strategies: (I) canonical activation; (II) split activation; and (III) steric-regulated split activation. (**B**–**D**) Real-time fluorescence kinetics for the three strategies. **(E)**  *S*/*N* analysis at 90 min. Data are presented as mean ± SD (*n* = 3).

However, when integrating the EDC and CRISPR/Cas12a systems into a single reaction vessel for a one-pot cascade, a pronounced kinetic mismatch arises: the *trans*-cleavage rate of Cas12a is far faster than the strand-displacement amplification rate of the EDC, leading to substantial signal crosstalk. To clearly illustrate this issue and demonstrate the solution, we systematically compared three Cas12a activation strategies (Fig. 4A). In the canonical activation strategy (Fig. 4A and I), a full-length single-stranded activator perfectly matching the EDC output is directly employed. Owing to the absence of kinetic control, trace amounts of nonspecific intermediates are sufficient to instantly trigger burst-phase Cas12a cleavage, resulting in severe background leakage and premature saturation of the detection signal before the EDC has accumulated meaningful output (Fig. 4B). In the regular split activation strategy (Fig. 4A-II), dividing the activator into two short strands reduces basal activity to some extent; however, the spontaneous hybridization kinetics of the split strands remain poorly matched to the slower EDC amplification process, yielding appreciable background signals nonetheless (Fig. 4C).

To fundamentally overcome this bottleneck, we developed a steric-regulated split activation strategy, guided by the steric modulation principles established earlier. The key concept is to introduce programmable terminal extensions onto the split activators, thereby installing a spatial barrier between the activator and the Cas12a complex and establishing a defined kinetic activation threshold for Cas12a. As shown in Fig. 4A-III, this strategy effectively reshapes the cascade reaction kinetics. During the early reaction stage, the steric obstruction introduced by the terminal extensions suppresses low-concentration nonspecific assembly of the activator–Cas12a complex, enabling the EDC to accumulate output without premature Cas12a activation. Only when the EDC cycle releases sufficiently high concentrations of output strands capable of overcoming the steric barrier does Cas12a become specifically activated. As demonstrated in Fig. 4D and E, this strategy markedly decreases background signals and enhances the signal-to-noise ratio (*S*/*N*, where *S* and *N* correspond to fluorescence intensities in the presence and absence of target, respectively), achieving near-leakage-free cascade detection and effectively resolving the kinetic mismatch. In Supplementary Figs S13–S17, we further screened combinations of fixed strands and extension designs. The results showed that the configuration employing fixed Pd-ss11_DE3 together with S1’ as the activator strand achieved the best performance, efficiently suppressing background while retaining high responsiveness to target-triggered signal generation. This design provides a new conceptual framework for the deep integration of programmable nucleic acid systems with CRISPR-based detection platforms.

### Performance of the sterically regulated one-pot cascade system

**Figure 5.**
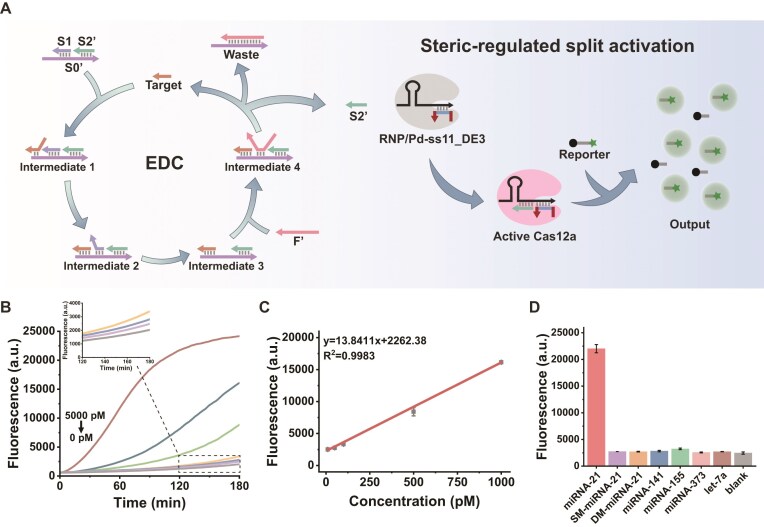
Working principle and analytical performance of the steric-regulated one-pot EDC–Cas12a cascade. (**A**) Schematic of the EDC integrating the sterically regulated split activator. (**B**) Real-time fluorescence kinetics across varying miRNA-21 concentrations (10 pM to 5 nM). (**C**) Linear regression analysis of fluorescence intensity versus miRNA-21 concentration (10 pM to 1 nM). (**D**) Specificity assessment against interfering sequences. Data are presented as mean ± SD (*n* = 3).

Based on the above findings, to further verify the reliability and practical value of the constructed cascaded system, we systematically evaluated its key performance metrics, including sensitivity and specificity. The detection principle is shown in Fig. 5A. The reaction mixture contains a pre-hybridized three-strand substrate complex (S0’–S1–S2’), a fuel strand (F’), and a Cas12a RNP complex preassembled with the sterically regulated split activator (Pd-ss11_DE3). In the absence of target, the terminal extension on the Pd strand imposes steric hindrance that kinetically locks Cas12a in an inactive state, thereby effectively suppressing background leakage. Upon the introduction of miRNA-21, the target initiates a toehold-mediated strand-displacement reaction with S0’, releasing S1, and exposing the previously concealed toehold on S0’. The fuel strand F’ subsequently drives the second displacement step, liberating both the recyclable TS and the output strand S2’. Accumulated S2’ functions as a critical unlocking species that cooperatively assembles with Pd-ss11_DE3 and overcomes the steric barrier, specifically activating Cas12a *trans*-cleavage and generating amplified fluorescence.

To achieve optimal analytical performance, we optimized the fuel strand sequence and RNP concentration (Supplementary Figs S18–S19). The results indicated that the F’4 fuel strand combined with 80 nM RNP yielded the highest *S*/*N* and was thus selected for subsequent assays. Under these optimized conditions, we next evaluated the system’s quantitative performance across a series of miRNA-21 concentrations. As shown in Fig. 5B, the fluorescence intensity increased progressively as the miRNA-21 concentration rose from 10 pM to 5 nM. Within the range of 10 pM to 1 nM, fluorescence intensity exhibited a strong linear correlation with target concentration (Fig. 5C), described by the regression equation *y* = 13.8411*x* + 2262.38, with a correlation coefficient of R^2^ = 0.9983. The limit of detection calculated based on the 3σ criterion was 1.24 pM [42, 43].

To evaluate the specificity of the strategy, we tested various interfering miRNAs (including miRNA-155, miRNA-373, miRNA-141, let-7a) as well as single-base mismatch (SM-miRNA-21) and double-base mismatch (DM-miRNA-21) sequences. As shown in Fig. 5D, only miRNA-21 induced a significant fluorescence increase, while the signals from other miRNAs and mismatch sequences were comparable to the blank control group. This indicates that the cascaded system has high specificity for the target miRNA-21.

Collectively, the sterically regulated one-pot cascade system exhibits picomolar-level sensitivity, a broad linear dynamic range, and single-nucleotide specificity, highlighting its potential application value in early disease biomarker detection and complex sample analysis, and facilitating the deep integration of programmable nucleic acid reaction networks with CRISPR technology.

## Conclusion

The immediate and uncontrolled activation of CRISPR nucleases often disrupts nucleic acid reaction networks and restricts their use to terminal modules, thereby limiting their applicability in temporally regulated or dynamically coordinated systems. This challenge is especially acute in contexts such as gene regulation, therapeutic delivery, or autonomous molecular computation, where delayed, gradual, or leakage-free activation of Cas12a is required. Addressing this unmet need, we developed a regulatory strategy based entirely on the structural engineering of split activators, enabling precise modulation of Cas12a activity without protein modification or external stimuli.

In this work, we established a steric-regulation framework in which rationally designed terminal extensions on split activators tune Cas12a *trans*-cleavage kinetics. By systematically dissecting the influence of extension orientation, length, and hybridization state, we identified quantitative and direction-specific rules that govern split-activator-mediated activation. These findings demonstrate that steric hindrance positioned at defined termini functions as a programmable kinetic barrier capable of modulating background leakage, assembly efficiency, and activation thresholds.

Leveraging these mechanistic insights, we integrated the sterically regulated split activator into an EDC to realize a fully one-pot cascade with Cas12a. The introduction of sequence-encoded steric barriers effectively suppresses premature or leakage-driven activation, enabling strict kinetic matching between the DNA circuit and Cas12a. The resulting system achieved a detection limit of 1.24 pM for miRNA-21, exhibited a linear response from 10 pM to 1 nM, and maintained high discrimination against single- and double-base mismatches, underscoring its robustness for nucleic acid diagnostics.

Beyond detection, the nucleic-acid-only and modular nature of the sterically tuned split activator provides a generalizable regulatory component for broader CRISPR applications. Its predictable activation thresholds and compatibility with nucleic acid circuitry support integration into CRISPR logic networks, spatiotemporally controlled gene regulation modules, microfluidic or implantable point-of-care testing (POCT) platforms, and intracellular CRISPR modulation schemes requiring delayed, graded, or background-free activation.

In conclusion, this study establishes steric engineering of split activators as a powerful and broadly applicable strategy for precise kinetic control of CRISPR/Cas12a. By elucidating the structure–function determinants underlying steric gating and demonstrating their direct translation into a high-performance one-pot cascade, we provide both mechanistic insight and a versatile regulatory toolkit that advances the controllability, programmability, and translational potential of CRISPR molecular systems.

## Supplementary Material

gkaf1535_Supplemental_File

## Data Availability

All data supporting the findings of this study are available within the article and its supplementary information or will be made available from the authors upon request.
