# Supplementary material for: Steric regulation of CRISPR/Cas12a trans-cleavage kinetics via split-activator extensions
Source: Nucleic Acids Res. 2026 Jan 14;54(2):gkaf1535. doi: 10.1093/nar/gkaf1535 (PMC12802953; doi:10.1093/nar/gkaf1535)
Supplement: gkaf1535_Supplemental_File [file gkaf1535_supplemental_file.pdf]

Supplementary materials

Steric regulation of CRISPR/Cas12a *trans*-cleavage kinetics via split activator extensions

Jianhong Zhang [a] †, Xin He [a] †, Jing Huang [b] †, Cheng Cheng [b], Guowei He [a], Ruili Xia [a], Jun Yang [c], Jianmei Chen [c], Lirong Guo [d], Debing Xiang [b], Feng Li [b], Jing Shi [e] *, Pu Li [a] *

[a] Department of Clinical Laboratory, Chongqing University Jiangjin Hospital, School of Medicine, Chongqing University, Chongqing, 402260, China

[b] Office of Hospital Administration, Chongqing University Jiangjin Hospital, School of Medicine, Chongqing University, Chongqing, 402260, China

[c] Department of ICU, Chongqing University Jiangjin Hospital, School of Medicine, Chongqing University, Chongqing, 402260, China

[d] Department of Pediatrics, Chongqing University Jiangjin Hospital, School of Medicine, Chongqing University, Chongqing, 402260, China

[e] Department of Laboratory Medicine, The First Affiliated Hospital of Chongqing Medical University, Chongqing, 400016, China.

* To whom correspondence should be addressed: Pu Li, lipu.cqu@cqu.edu.cn; Correspondence may also be addressed to Jing Shi, jingyuns@hospital.cqmu.edu.cn.

† The first three authors should be regarded as Joint First Authors.

Table S1. The oligonucleotide sequences used in this work

Name	Sequence (5'-3')
crRNA	UAAUUUCUACUAAGUGUAGAUACCCCU AUCACCUCGACUCC
TS	GGAGTCGAGGTGATAGGGGT
Reporter	***FAM***-TTATT-***BHQ1***
Pd-ss6	GGAGTC
Pd-ss7	GGAGTCG
Pd-ss8	GGAGTCGA
Pd-ss9	GGAGTCGAG
Pd-ss10	GGAGTCGAGG
Pd-ss11	GGAGTCGAGGT
Pd-ss12	GGAGTCGAGGTG
Pd-ss13	GGAGTCGAGGTGA
Pd-ss14	GGAGTCGAGGTGAT
Pp-ss6	AGGGGT
Pp-ss7	TAGGGGT
Pp-ss8	ATAGGGGT
Pp-ss9	GATAGGGGT
Pp-ss10	TGATAGGGGT
Pp-ss11	GTGATAGGGGT

Pp-ss12	GGTGATAGGGGT
Pp-ss13	AGGTGATAGGGGT
Pp-ss14	GAGGTGATAGGGGT
Pd-ss11-c	ACCTCGACTCC
Pp-ss9-c	ACCCCTATC
Pd-ss11_5'E3	ACAGGAGTCGAGGT
Pd-ss11_3'E3	GGAGTCGAGGTACA
Pd-ss11_DE3	ACAGGAGTCGAGGTACA
Pd-ss11_5'E6	CACACAGGAGTCGAGGT
Pd-ss11_3'E6	GGAGTCGAGGTACACAC
Pd-ss11_DE6	CACACAGGAGTCGAGGTACACAC
Pd-ss11_5'E9	ACACACACAGGAGTCGAGGT
Pd-ss11_3'E9	GGAGTCGAGGTACACACACA
Pd-ss11_DE9	ACACACACAGGAGTCGAGGTACACACAC A
Pd-ss11_5'E12	CACACACACACAGGAGTCGAGGT
Pd-ss11_3'E12	GGAGTCGAGGTACACACACACAC
Pd-ss11_DE12	CACACACACACAGGAGTCGAGGTACACA CACACAC
Pd-ss11_5'E3-c	TGTACCTCGACTCC
Pd-ss11_3'E3-c	ACCTCGACTCCTGT

Pd-ss11_DE3-c	TGTACCTCGACTCCTGT
Pd-ss11_5’E6-c	GTGTGTACCTCGACTCC
Pd-ss11_3’E6-c	ACCTCGACTCCTGTGTG
Pd-ss11_DE6-c	GTGTGTACCTCGACTCCTGTGTG
Pd-ss11_5’E9-c	TGTGTGTGTACCTCGACTCC
Pd-ss11_3’E9-c	ACCTCGACTCCTGTGTGTGT
Pd-ss11_DE9-c	TGTGTGTGTACCTCGACTCCTGTGTGTGT
Pd-ss11_5’E12-c	GTGTGTGTGTGTACCTCGACTCC
Pd-ss11_3’E12-c	ACCTCGACTCCTGTGTGTGTGTG
Pd-ss11_DE12-c	GTGTGTGTGTGTACCTCGACTCCTGTGTG TGTGTG
Pp-ss9_5’E3	CACGATAGGGGT
Pp-ss9_3’E3	GATAGGGGTCAC
Pp-ss9_DE3	CACGATAGGGGTCAC
Pp-ss9_5’E6	ACACACGATAGGGGT
Pp-ss9_3’E6	GATAGGGGTCACACA
Pp-ss9_DE6	ACACACGATAGGGGTCACACA
Pp-ss9_5’E9	CACACACACGATAGGGGT
Pp-ss9_3’E9	GATAGGGGTCACACACAC
Pp-ss9_DE9	CACACACACGATAGGGGTCACACACAC
Pp-ss9_5’E12	ACACACACACACGATAGGGGT

Pp-ss9_3’E12	GATAGGGGTCACACACACACA
Pp-ss9_DE12	ACACACACACACGATAGGGGTCACACAC ACACA
Pp-ss9_5’E3-c	GTGACCCCTATC
Pp-ss9_3’E3-c	ACCCCTATCGTG
Pp-ss9_DE3-c	GTGACCCCTATCGTG
Pp-ss9_5’E6-c	TGTGTGACCCCTATC
Pp-ss9_3’E6-c	ACCCCTATCGTGTGT
Pp-ss9_DE6-c	TGTGTGACCCCTATCGTGTGT
Pp-ss9_5’E9-c	GTGTGTGTGACCCCTATC
Pp-ss9_3’E9-c	ACCCCTATCGTGTGTGTG
Pp-ss9_DE9-c	GTGTGTGTGACCCCTATCGTGTGTGTG
Pp-ss9_5’E12-c	TGTGTGTGTGTGACCCCTATC
Pp-ss9_3’E12-c	ACCCCTATCGTGTGTGTGTGT
Pp-ss9_DE12-c	TGTGTGTGTGTGACCCCTATCGTGTGTGT GTGT
Pp-ss9_DE6-cPAM	ACACACGATAGGGGTTAAACA
Pp-ss9_DE6-cPAM-c	TGTTTAACCCCTATCGTGTGT
Pp-ss9_DE6-ncPAM1	ACACACGATAGGGGTCATACA
Pp-ss9_DE6-ncPAM1-c	TGTATGACCCCTATCGTGTGT
Pp-ss9_DE6-ncPAM2	ACACACGATAGGGGTGACACA

Pp-ss9_DE6-ncPAM2-c	TGTGTCACCCCTATCGTGTGT
S0	TCAACATCAGTCTGATAAGCTATAGGGACCCCTATCACCTCGACTCC
S1	CCCTATAGCTTATCAGACT
S2	GGAGTCGAGGTGATAGGGGT
F	GGAGTCGAGGTGATAGGGGTCCCTATAGCTTATCAGACT
S0’	TCAACATCAGTCTGATAAGCTATAGGGGTGACCCCTATCGTG
S2’	CACGATAGGGGTCAC
F’(F’2)	TAGGGGTCACCCCTATAGCTTATCAGACT
Alt-S0’	TCAACATCAGTCTGATAAGCTATAGGGTACCTCGACTCCT
Alt-S2’	AGGAGTCGAGGTA
Alt-F’	TCGAGGTACCCTATAGCTTATCAGACT
F’1	ATAGGGGTCACCCCTATAGCTTATCAGACT
F’3	AGGGGTCACCCCTATAGCTTATCAGACT
F’4	GGGGTCACCCCTATAGCTTATCAGACT
F’5	GGGTCACCCCTATAGCTTATCAGACT
miRNA-21	UAGCUUAUCAGACUGAUGUUGA
SM-miRNA-21	UAGCUUAUCACACUGAUGUUGA

DM-miRNA-21	UAGGUUAUCACACUGAUGUUGA
miRNA-155	UUAAUGCUAAUCGUGAUAGGGGUU
miRNA-373	GAAGUGCUUCGAUUUUGGGGUGU
miRNA-141	UAACACUGUCUGGUAAAGAUGG
let-7a	UGAGGUAGUAGGUUGUAUAGUU

Note: c, complementary; cPAM, canonical PAM; ncPAM, non-canonical PAM; Alt, Alternative; SM, single-base mismatch; DM, double-base mismatch; red marker, mismatch site; 5’E, 3’E, and DE denote extensions at the 5’ end, 3’ end, and both ends, respectively; numeric suffix, extension length (nt); ss and ds denote single-stranded and double-stranded forms, respectively.

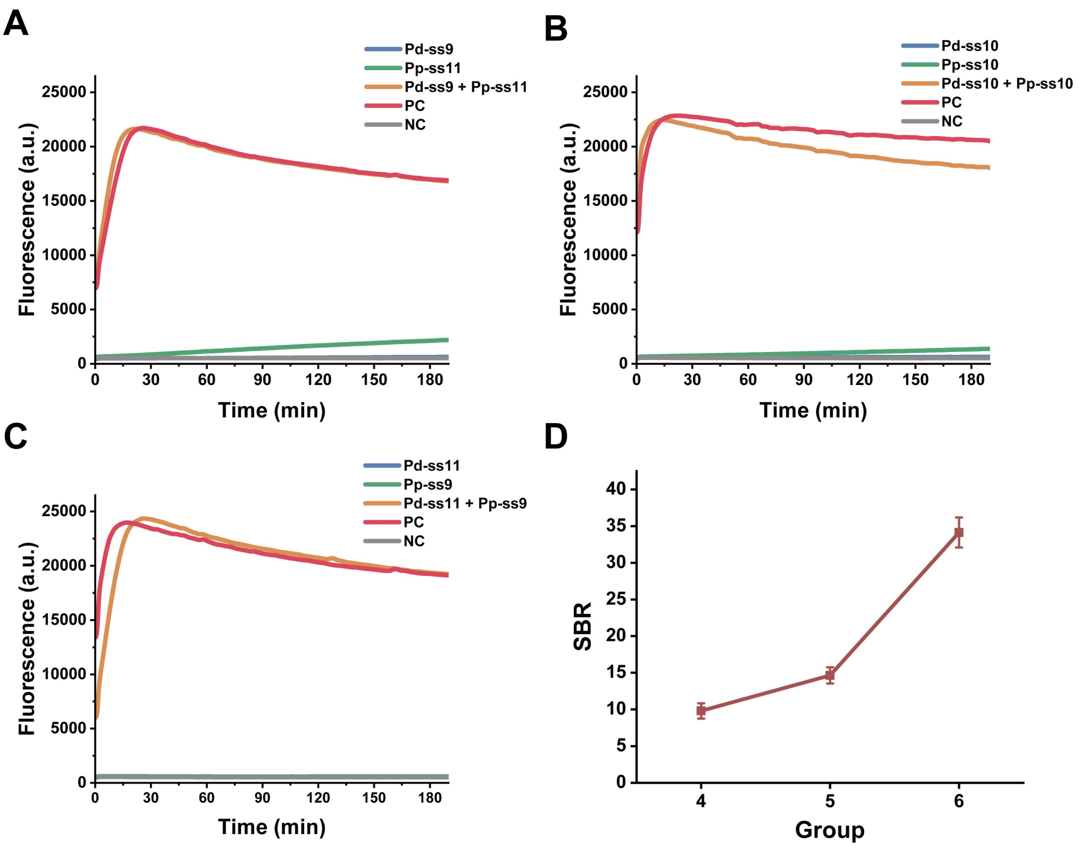

Fig. S1. Long-term stability of selected split activators. Real-time fluorescence kinetics for (A) Group 4, (B) Group 5, and (C) Group 6, together with (D) SBR analysis at 60 min. Data are presented as mean ± SD (n = 3).

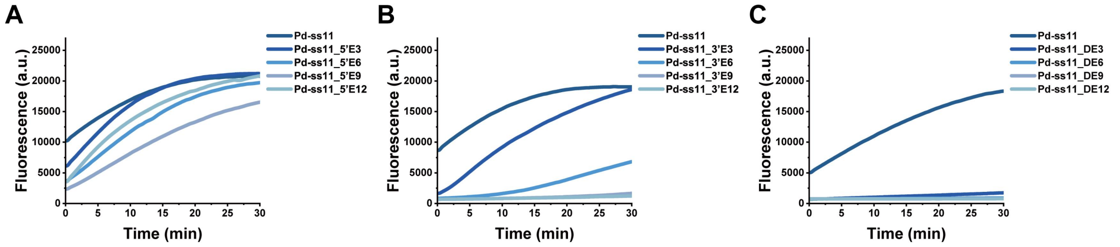

Fig. S2. Real-time fluorescence kinetics with fixed Pp-ss9 and Pd-ss11 bearing ssDNA extensions at the (A) 5'-end, (B) 3'-end, or (C) dual ends.

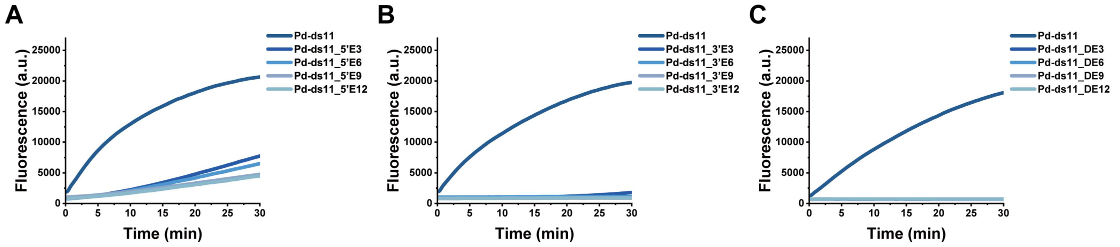

Fig. S3. Real-time fluorescence kinetics with fixed Pp-ss9 and Pd-ds11 bearing rigid dsDNA extensions at the (A) 5'-end, (B) 3'-end, or (C) dual ends.

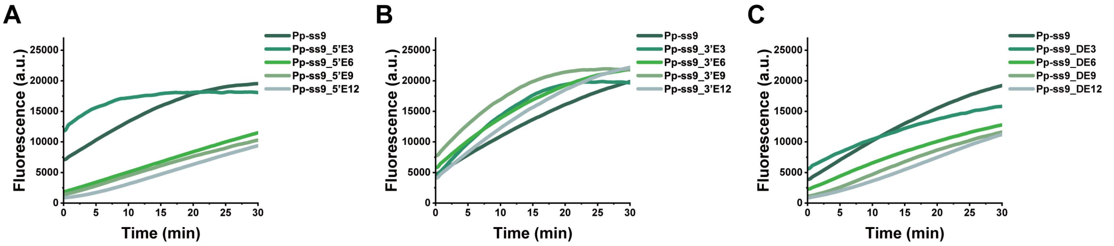

Fig. S4. Real-time fluorescence kinetics with fixed Pd-ss11 and Pp-ss9 bearing ssDNA extensions at the (A) 5'-end, (B) 3'-end, or (C) dual ends.

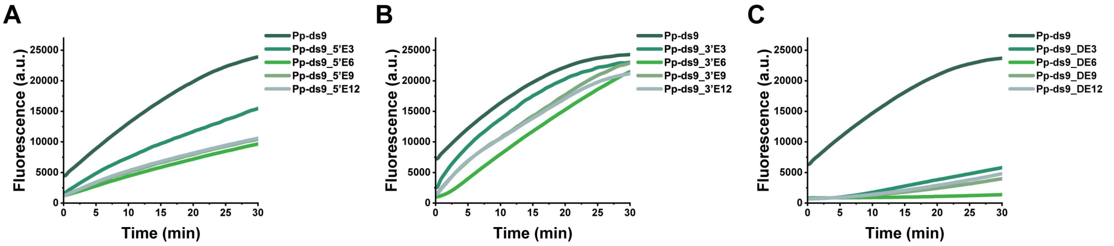

Fig. S5. Real-time fluorescence kinetics with fixed Pd-ss11 and Pp-ds9 bearing rigid dsDNA extensions at the (A) 5'-end, (B) 3'-end, or (C) dual ends.

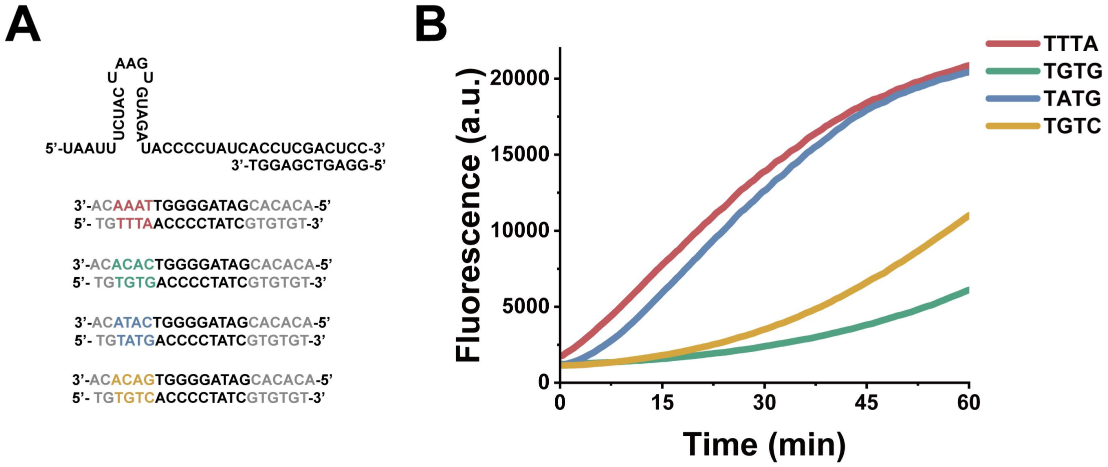

Fig. S6. Robustness of steric regulation across PAM sequences. (A) Schematic of the Pd-ss11 + Pp-ds9_DE6 system with different PAMs (TTTA, TGTG, TATG, TGTC). (B) Real-time fluorescence kinetics for the four PAM sequences.

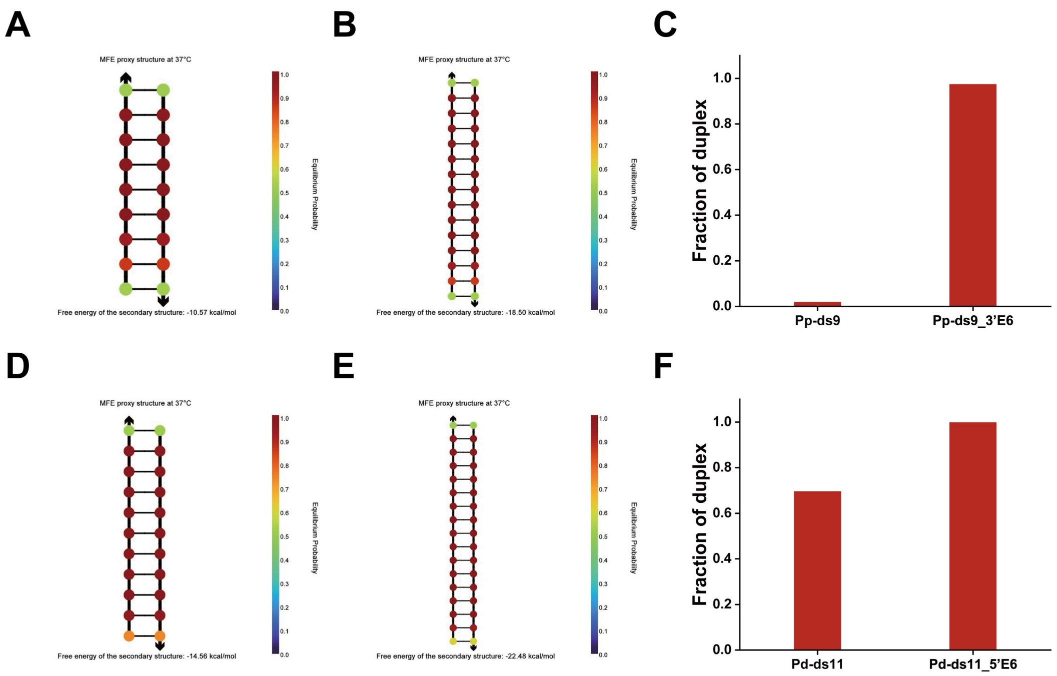

Fig. S7. Thermodynamic stability analysis of dsDNA anchors (NUPACK, 37°C). Stability comparison for the Pp anchor: (A) Pp-ds9; (B) Pp-ds9_3'E6; (C) Comparison of hybridization fraction. Stability comparison for the Pd anchor: (D) Pd-ds11; (E) Pd-ds11_5'E6; (F) Comparison of hybridization fraction.

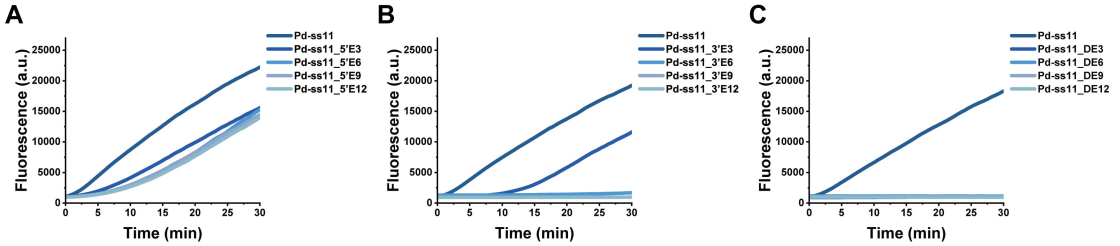

Fig. S8. Real-time fluorescence kinetics with fixed Pp-ds9 and Pd-ss11 bearing ssDNA extensions at the (A) 5'-end, (B) 3'-end, or (C) dual ends.

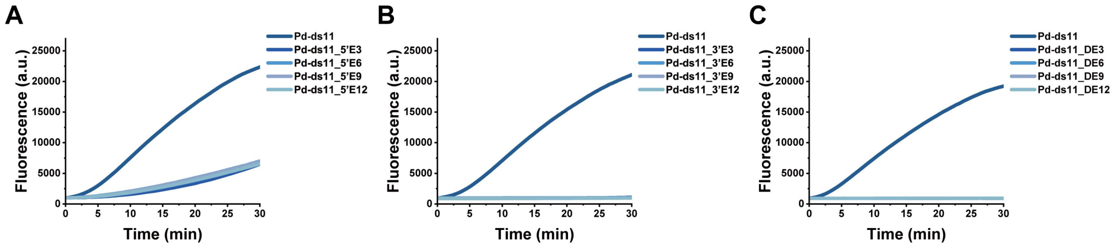

Fig. S9. Real-time fluorescence kinetics with fixed Pp-ds9 and Pd-ds11 bearing rigid dsDNA extensions at the (A) 5'-end, (B) 3'-end, or (C) dual ends.

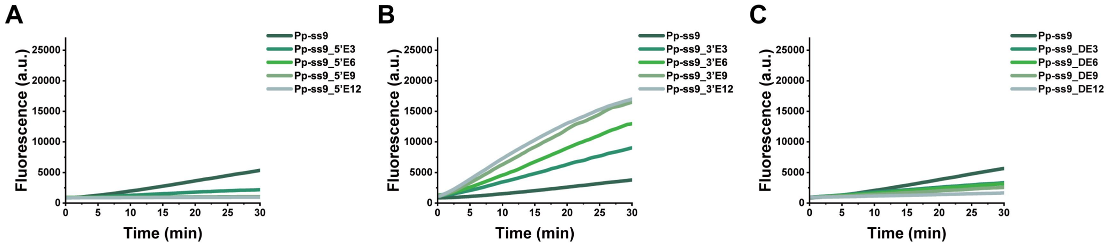

Fig. S10. Real-time fluorescence kinetics with fixed Pd-ds11 and Pp-ss9 bearing ssDNA extensions at the (A) 5'-end, (B) 3'-end, or (C) dual ends.

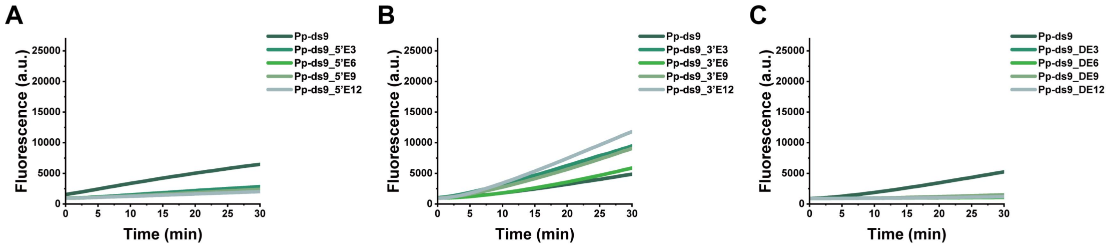

Fig. S11. Real-time fluorescence kinetics with fixed Pd-ds11 and Pp-ds9 bearing rigid dsDNA extensions at the (A) 5'-end, (B) 3'-end, or (C) dual ends.

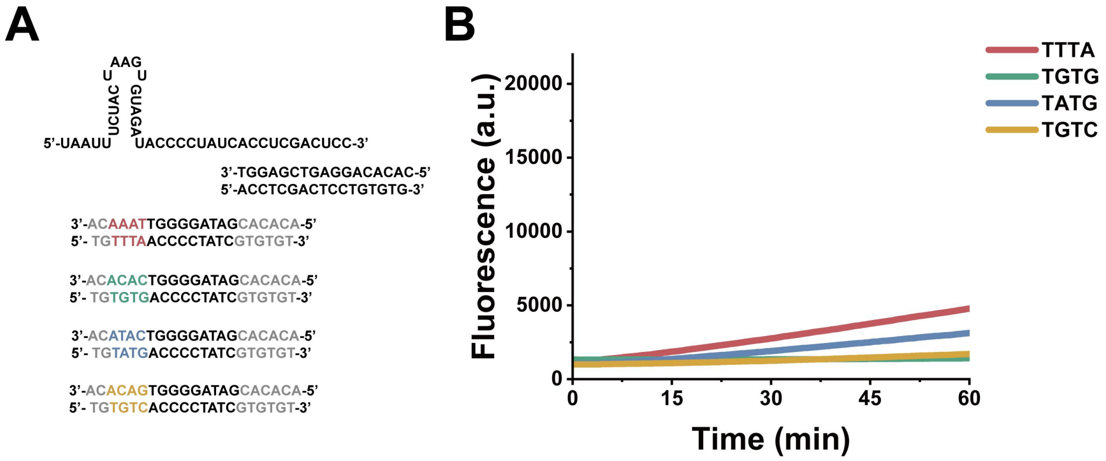

Fig. S12. Universality of dual-rigid steric amplification across PAM sequences. (A) Schematic of the Pd-ds11_5'E6 + Pp-ds9_DE6 system with different PAMs. (B) Real-time fluorescence kinetics for the four PAM sequences.

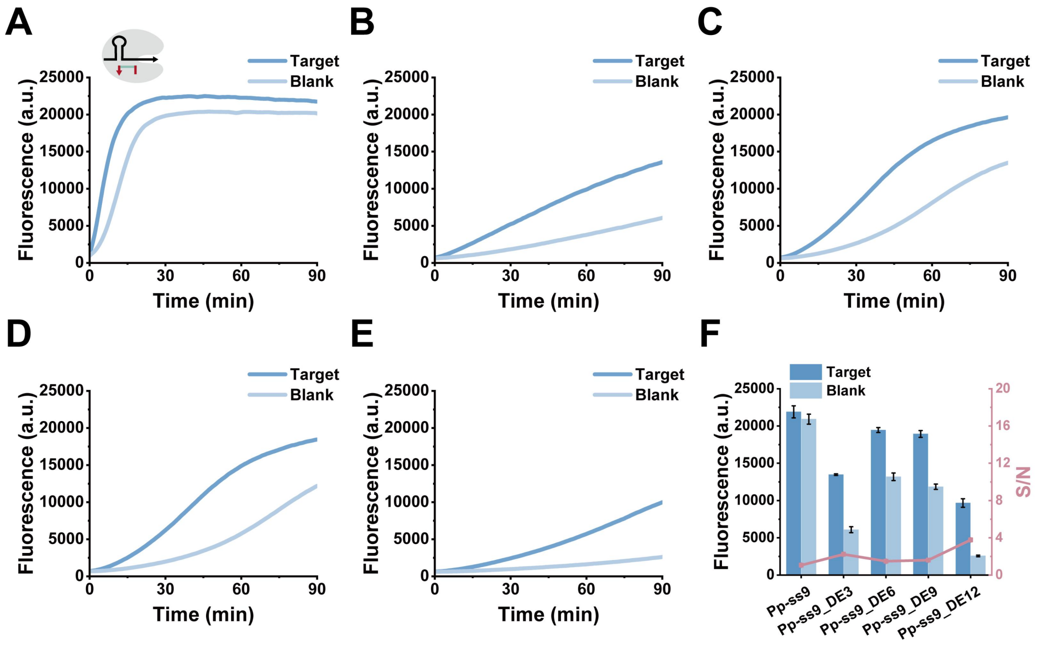

Fig. S13. Steric-regulated strategies for the one-pot EDC–Cas12a cascade using a fixed Alt-S2' split activator and Pp-ss9 bearing dual-end extensions (DE0–DE12). (A–E) Real-time fluorescence kinetics for Pp-ss9 variants (DE0, DE3, DE6, DE9, DE12). (F) S/N ratio comparison at 90 min.

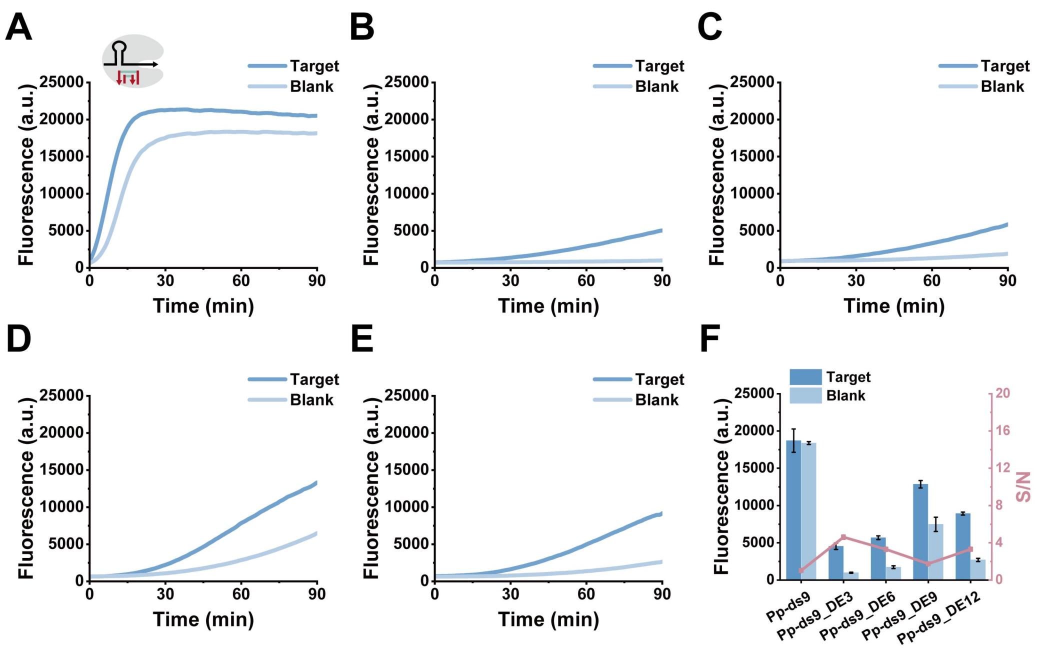

Fig. S14. Steric-regulated strategies for the one-pot EDC–Cas12a cascade using a fixed Alt-S2' split activator and Pp-ds9 bearing dual-end extensions (DE0–DE12). (A–E) Real-time fluorescence kinetics for Pp-ds9 variants (DE0, DE3, DE6, DE9, DE12). (F) S/N ratio comparison at 90 min.

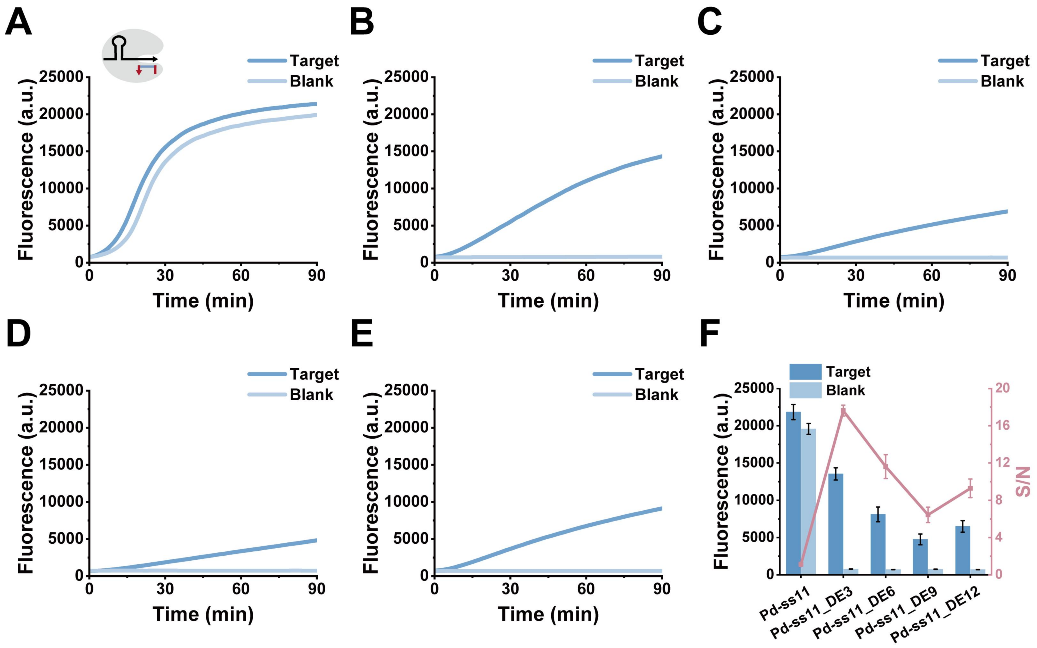

Fig. S15. Steric-regulated strategies for the one-pot EDC–Cas12a cascade using a fixed S2' split activator and Pd-ss11 bearing dual-end extensions (DE0–DE12). (A–E) Real-time fluorescence kinetics for Pd-ss11 variants (DE0, DE3, DE6, DE9, DE12). (F) S/N ratio comparison at 90 min.

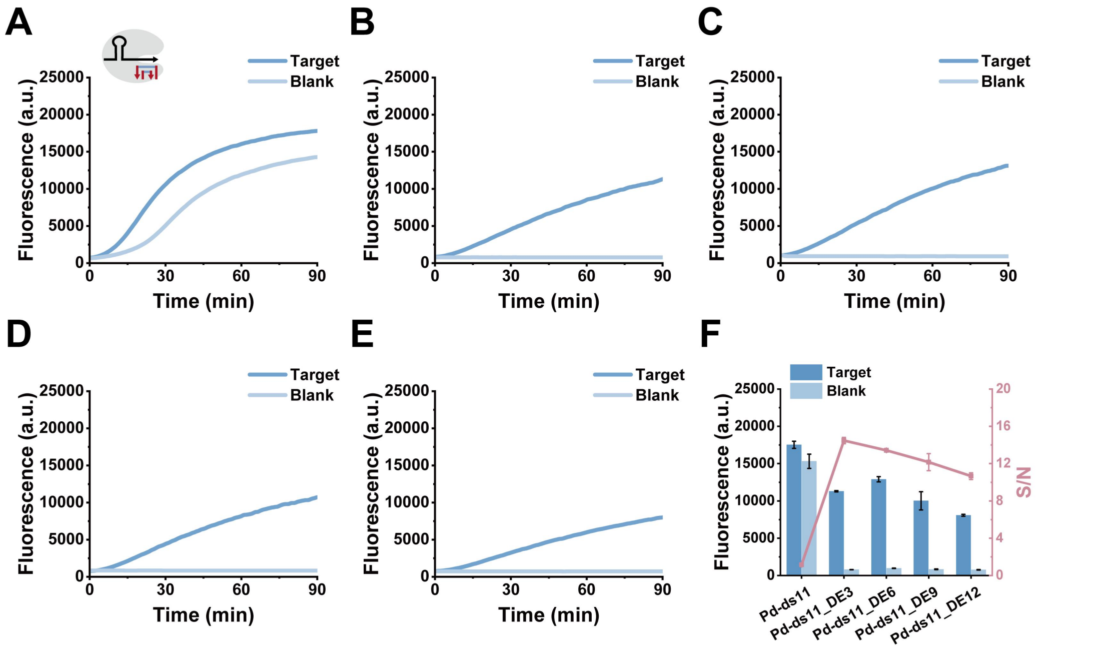

Fig. S16. Steric-regulated strategies for the one-pot EDC–Cas12a cascade using a fixed S2' split activator and Pd-ds11 bearing dual-end extensions (DE0–DE12). (A–E) Real-time fluorescence kinetics for Pd-ds11 variants (DE0, DE3, DE6, DE9, DE12). (F) S/N ratio comparison at 90 min.

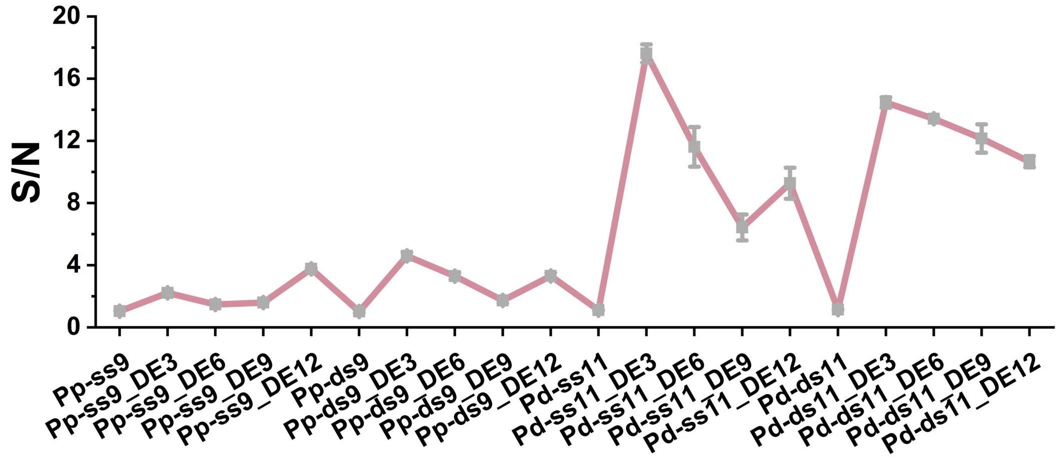

Fig. S17. Comprehensive S/N comparison for all screened cascade strategies.

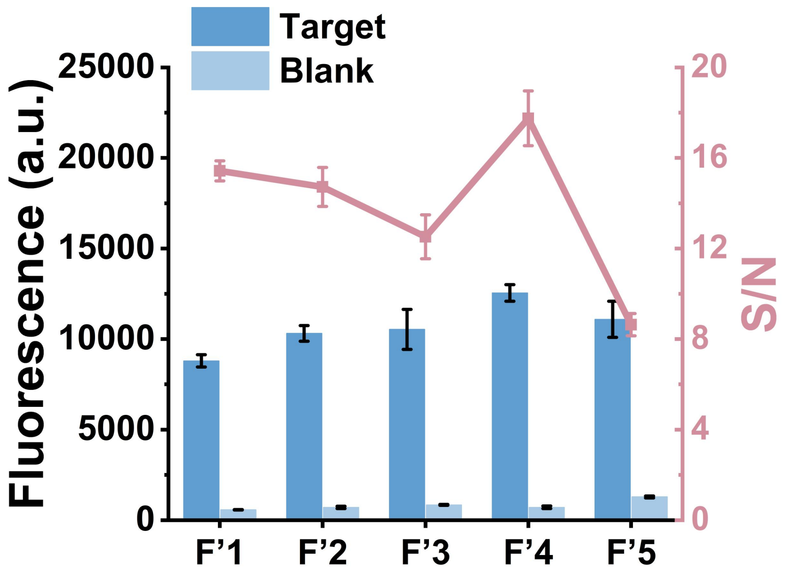

Fig. S18. Optimization of F' sequence. Data are presented as mean ± SD (n = 3).

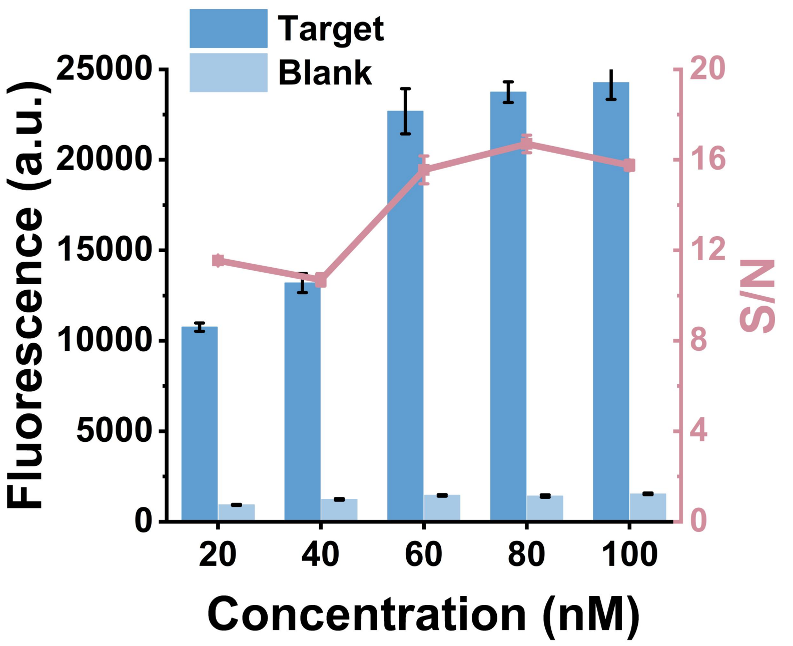

Fig. S19. Optimization of RNP concentration. Data are presented as mean ± SD (n = 3).
